# Potential Impact of a Free Online HIV Treatment Response Prediction System for Reducing Virological Failures and Drug Costs after Antiretroviral Therapy Failure in a Resource-Limited Setting

**DOI:** 10.1155/2013/579741

**Published:** 2013-09-24

**Authors:** Andrew D. Revell, Gerardo Alvarez-Uria, Dechao Wang, Anton Pozniak, Julio S. Montaner, H. Clifford Lane, Brendan A. Larder

**Affiliations:** ^1^The HIV Resistance Response Database Initiative (RDI), 14 Union Square, London N1 7DH, UK; ^2^Rural Development Trust (RDT) Hospital, Bathalapalli, 515661 AP, India; ^3^Chelsea and Westminster Hospital, London SW10 9NH, UK; ^4^BC Centre for Excellence in HIV/AIDS, Vancouver, Canada; ^5^National Institute of Allergy and Infectious Diseases, Bethesda, MD 20892, USA

## Abstract

*Objective*. Antiretroviral drug selection in resource-limited settings is often dictated by strict protocols as part of a public health strategy. The objective of this retrospective study was to examine if the HIV-TRePS online treatment prediction tool could help reduce treatment failure and drug costs in such settings. *Methods*. The HIV-TRePS computational models were used to predict the probability of response to therapy for 206 cases of treatment change following failure in India. The models were used to identify alternative locally available 3-drug regimens, which were predicted to be effective. The costs of these regimens were compared to those actually used in the clinic. *Results*. The models predicted the responses to treatment of the cases with an accuracy of 0.64. The models identified alternative drug regimens that were predicted to result in improved virological response and lower costs than those used in the clinic in 85% of the cases. The average annual cost saving was $364 USD per year (41%). *Conclusions*. Computational models that do not require a genotype can predict and potentially avoid treatment failure and may reduce therapy costs. The use of such a system to guide therapeutic decision-making could confer health economic benefits in resource-limited settings.

## 1. Introduction

Combination antiretroviral therapy (ART) has the potential to turn HIV infection into a chronic manageable condition. However, the roll-out of ART is more difficult in resource-limited settings (RLS) due to the relatively high cost of drugs and diagnostics, and the lack of infrastructure and clinical expertise. To enable the rapid scale-up of ART, many national programs in RLS adopted the public health approach advocated by the World Health Organization (WHO) of using fixed ART regimens with little choice of drug regimen for first line or salvage following treatment failure. Moreover, due to economic constraints, failure is usually detected using clinical criteria or CD4 lymphocyte counts, rather than viral load monitoring [1]. This strategy has been shown to be associated with deferred treatment switching, accumulation of resistance, and increased morbidity and mortality [2–7]. This scenario is in stark contrast to well-resourced settings in which treatment failure is detected earlier as a result of routine viral load monitoring and ART regimens which are tailored according to individual characteristics of the patients such as patient's preferences, drug interactions with other medications, cardiovascular risk factors, and other comorbidities, as well as HIV drug resistance. A genotypic resistance test is, therefore, generally performed and interpreted by experts who select a combination of drugs from the 25 or so available to tailor therapy for the individual patient [8]. 

The HIV Resistance Response Database Initiative (RDI) has developed computational models that use genotype, viral load, CD4 count, and treatment history variables to predict the response to a new drug combination following virological failure with approximately 80% accuracy [9–11]. The models have been used to power a free experimental web-based HIV treatment response prediction system (HIV-TRePS) assessed by experienced HIV physicians in two clinical pilot studies as a useful aid to clinical practice [12]. 

However, genotypic resistance testing is relatively expensive and requires sophisticated infrastructure and scientific expertise that are not readily available in many RLS. Thus, alternative models have been developed that do not require a genotype but rely on CD4 counts, viral loads, and treatment history for their predictions. This has resulted in only a small loss of performance to a level of accuracy at least comparable to that of using genotypic sensitivity scores (from genotyping with rules-based interpretation) as a predictor of response [11, 13–15]. These models are able to predict most of the cases where the salvage regimen selected in the clinic failed and are also able to identify alternative regimens comprising locally available drugs that are predicted to be effective [16, 17]. However, not all ART combinations can be afforded in RLS. In this study, we analysed the potential impact of the system to improve treatment decision making of clinicians in an RLS who had to initiate salvage therapy for patients with ART failure, without increasing the cost of the treatment. Specifically, we asked the following question: is the system able to identify alternative regimens that are predicted to be more effective and less costly than the regimen that was used and failed in clinics in an RLS?

## 2. Methods

### 2.1. Computational Models

The models used for this study were the eleven random forest models in use to power the on-line HIV Treatment Prediction Tool, HIV-TRePS (v3.3.1.0) for cases without a genotype. These models were developed and validated during 2011 using methodology described in detail elsewhere [10, 11]. The models were trained to estimate the probability of virological response, defined as a follow-up plasma viral load of less than 400 copies of HIV RNA/mL, this being the lower limit of detection of some assays in use at the time that the data were collected by the various collaborating clinics. 

The models made their predictions based on the following variables: baseline viral load value (while on the previous failing therapy, no more than 8 weeks before therapy change), baseline CD4 count (no more than 8 weeks before therapy change), treatment history (antiretroviral drugs to which the patient has been exposed in the past), the drugs in the new treatment and the time to follow-up. These data from almost 16,000 treatment change episodes (TCEs), collected from clinical practice, were partitioned at random and used to train (*n* = 14,891) and test (*n* = 800) the models. These data came principally from North America, Western Europe, Australia, and Japan and did not include any data from RLS. The accuracy of the models was assessed primarily using the area under the receiver-operator curve (AUROC), which takes into account both sensitivity and specificity. During testing with the independent test set of 800 TCEs, the operating point (OOP) of the models in classifying their predictions as response or failures to maximise sensitivity and specificity was established.

### 2.2. Testing the Models with Data from an RLS

The RF models were tested for potential utility with a number of datasets from RLS, including 206 TCEs from an HIV cohort study in the district of Anantapur, India. The characteristics of this cohort have been described elsewhere [18, 19]. Firstly, in order to assess the accuracy of the models, the baseline data from the Indian TCEs were used to obtain predictions of response or failure which were then compared to the responses (follow-up viral loads) observed in the clinic. 

Secondly, in order to assess the potential of the models to help avoid treatment failure, they were used to perform “in silico” modelling of alternative antiretroviral regimens to identify alternatives that were more likely to produce virological response. The baseline data were used by the models to make predictions of response for alternative three-drug regimens comprising combinations of the 10 drugs that were locally available: zidovudine, didanosine, lamivudine, abacavir, tenofovir, nevirapine, lopinavir, atazanavir, nelfinavir, and ritonavir as a booster for the protease inhibitors (PIs)—all PIs used other than nelfinavir were boosted with ritonavir. Stavudine was also used in India at the time but was excluded from the analysis as the use of this drug is no longer recommended, for toxicity reasons. We identified alternative regimens that met the following criteria: (a) virological response was predicted (the estimated probability of response was above the OOP for the models) and (b) the estimated probability of response was higher than for the regimen actually used in the clinic.

Finally, the annual costs of these drugs (including the available coformulations) were used to compare the annual cost of the actual salvage regimens used in the clinic with the costs of the alternative regimens identified by the models (Table 2).

## 3. Results

### 3.1. Characteristics of the Datasets

The Indian TCEs occurred between 2007 and 2012, with 198 (96%) from 2008 to 2010. The baseline, treatment, and response characteristics of the datasets are summarised in Table 1. Males outnumbered females in the RDI data sets by around 5 : 1, whereas there was a gender balance in the Indian data. The Indian patients were younger than those from the RDI data sets (median age = 28 versus 39) and had somewhat higher baseline viral loads (median of 4.79log⁡_10_ copies/mL versus 3.77log⁡_10_ copies/mL for the training data). This is consistent with patients in RLS switching after a greater degree of virological failure. All the cases from India had received NRTIs and NNRTIs in their history, with 6% having experience of protease inhibitors (PIs). Accordingly, 54% of the Indian cases had been switched onto 2 NRTIS + PI. The remaining Indian cases had been switched onto 3N(t)RTIs and a PI, all but two of which included TDF.

### 3.2. Accuracy of the Models

On testing with the 800 independent cases from the same settings as the training data, the models achieved an AUROC of 0.77 (95% CI 0.74, 0.80), where 1.00 would be perfect accuracy. Sensitivity was 71% (95% CI 67%, 76%) and specificity was 72% (95% CI 67%, 77%). When the RF models were used to predict the response to the salvage therapy in the Indian cases, the AUC was 0.64 (95% CI 0.57, 0.72), with a sensitivity of 55% (95% CI 47%, 65%) and specificity of 67% (95% CI 60%, 79%).

The models were able to identify an alternative regimen that was predicted to produce virological response, comprising only locally available drugs, in all 206 cases (Table 3). Of all the alternative regimens that were predicted to be effective, we found alternative regimens with a higher estimated probability of response than the regimen used in the clinic for 175 (85%) of the cases and for 55 (88%) of the 74 cases where the new regimen used in the clinic failed.

The models were able to identify one or more alternative regimens that were predicted to produce virological response with a lower annual cost than the regimen selected in the clinic for all these cases. The mean number of cost-saving alternative regimens predicted to produce virological response was 10 overall and 8 for those cases that failed in the clinic. The mean cost saving of the median cost alternative in each case was $364 USD per year (95% CI: $332, $395), a mean percentage cost reduction of 41% (95% CI: 38%, 45%). Considering only those cases where the salvage regimen introduced in the clinic failed, the mean cost saving was $421 USD per year (95% CI: $361, $481), a percentage reduction in cost of 45% (95% CI: 38%, 51%). 

Taking the least expensive of the alternative regimens that were predicted to be effective in each case produced an average cost saving of $555 USD per year (95% CI: $509, $601), a mean percentage reduction of 63% (95% CI: 61%, 65%). For the failures, the mean cost saving from the least expensive alternative was $638 USD per year, (95% CI: $554, $723), a percentage reduction of 68% (95% CI: 64%, 71%).

## 4. Discussion

The computational models predicted virological response to salvage therapy in India, without the results of genotypic resistance testing, with a degree of accuracy that is encouraging. While the models were not as accurate for Indian cases as they were with cases from the countries that provided the training data, a phenomenon seen in previous studies, their predictive accuracy was comparable to that seen historically from genotyping with rules-based interpretation [11]. 

The models were able to identify alternative regimens with higher estimated probability of response and lower cost than the actual regimen used in the clinic in 85% of the cases. This suggests that, had physicians been able to use the system to assist their treatment decisions, the number of virological failures could have been reduced. Furthermore, the use of the models could also potentially reduce the cost of therapy substantially, suggesting that the system has considerable clinical and health economic utility.

The study has some limitations. Firstly, it was retrospective and, as such, no firm claims can be made for the clinical benefit that the use of the system as a treatment support tool could provide, which should ideally be investigated via a randomized prospective trial. 

The RDI's relative shortage of complete TCEs that include plasma viral loads from RLS, including India, meant that the test set was relatively modest. With inclusion of more TCEs from RLS in the future, the accuracy of the models to predict response to salvage therapy in RLS will probably increase, a prediction that has been borne out by initial results from new models including data from RLS [20]. 

The system requires viral load for estimating the response to the ART regimens. Although viral load monitoring is not yet widely available in RLS, it is now recommended as the preferred approach to monitoring ART success and diagnosing treatment failure in the latest WHO guidelines [21]. As more affordable viral load technologies become available, viral load monitoring is becoming more commonplace in RLS.

The treatment history of the Indian cases was relatively simple with most moving from first line therapy involving two nucleoside analogues and a non-nucleoside analogue reverse transcriptase inhibitors onto two NRTIs and a protease inhibitor. Models such as these are likely to be of more utility in more complex cases and this warrants further study. 

Finally, the current costs of the drugs were used for this retrospective study since it was not possible to determine with certainty the cost and the availability of all drugs included in the models at the time of the treatment decision. While this may have affected the analysis somewhat, the fact that the models were able to identify several alternative regimens that were predicted to be effective and were less costly than the regimen used for the great majority of the cases suggests that this phenomenon is robust. Moreover, one of the most important advantages of the HIV-TRePS system over a rigid public health approach is the capacity of the system to provide information about the cost and predicted effectiveness of a wide range of ART regimen options. With the HIV-TRePS system, the treating clinicians can select from among these ART regimens taking into account the characteristics of the patient (including other comorbidities, previous adherence problems, and nonART medication) with the characteristics of the ART regimens (including side effects, number of tablets, and drug interactions) ordered by probability of response and cost. 

## 5. Conclusions 

This study demonstrates that computational models can predict virological response to antiretroviral therapy without a genotype with encouraging accuracy in an RLS. The use of these models can potentially help clinicians in RLS to reduce the number of treatment failures by identifying effective alternatives and may reduce the cost of drug therapy. These results suggest that the use of this freely available system to guide therapeutic decision making could confer health economic benefits in regions where cost-effective solutions to HIV management are of paramount importance.

## Figures and Tables

**Table 1:** Characteristics of the treatment change episodes in the training and test sets.

	Training set	RDI test set	India
TCEs (*n*)	14,891	800	206
Patients (*n*)	4,878	800	168
Male	11,006	601	109
Female	2,341	137	97
Median age	40	42	28
Baseline data (*n*)			
Median baseline VL (log_10_ c/mL)	3.77	3.79	4.79
Mean baseline VL (log_10_ c/mL)	3.74	3.74	4.75
Range of baseline VL (log_10_ c/mL)	1.71–7.0	1.71–6.01	2.96–6.82
Median baseline CD4 (cells/*μ*L)	260	260	274
Mean baseline CD4 (cells/*μ*L)	310	294	357
Treatment history (*n*)			
Number of previous drugs (median)	5	5	3
NRTI experience (%)	100%	100%	100%
NNRTI experience (%)	68%	67%	100%
PI experience (%)	83%	81%	6%
Failures (>2.6 log_10_ c/mL follow-up VL) (*n*)	6,501	309	74
Percent	44%	39%	36%
Responses (*n*)	8,390	491	132
Percent	56%	61%	64%
New Regimens (*n*)			
2NRTI + PI (%)	35%	35%	54%
2NRTI + NNRTI (%)	24%	22%	0%
3NRTIs + PI (%)	17%	19%	46%
Other (%)	24%	43%	0%

TCEs: treatment change episodes. VL: viral load. NRTI: nucleoside reverse transcriptase inhibitor. NNRTI: non-nucleoside reverse transcriptase inhibitor. PI: protease inhibitor.

**Table 2:** Annual costs of drugs and coformulations available to the Indian cohort.

	Annual cost (rupees)	Annual cost ($)*
Zidovudine	4,168.30	79.23
Lamivudine	2,357.90	44.82
Didanosine	3,615.20	68.72
Abacavir	12,634.84	240.16
Tenofovir	4,380.00	83.26
Nevirapine	3,197.40	60.78
Efavirenz	4,055.15	77.08
Lopinavir/ritonavir	30,127.10	572.66
Atazanavir	7,647.97	145.37
Ritonavir (atazanavir booster)	4,055.52	77.09
Nelfinavir	61,320.00	1,165.57
Coformulations		
Zidovudine + lamivudine 300 : 150 mg	5,416.60	102.96
Tenofovir + lamivudine 300 : 300 mg	4,732.83	89.96
Tenofovir + emtricitabine 300 : 200 mg	7,300.00	138.76

*1 Indian rupee = 0.019008 U.S. dollars.

**Table 3:** Results of in silico modelling of alternative regimens.

Analysis	Sample	
	All (*n* = 206)	Failures (*n* = 74)
(1) No. (%) of cases for which the models were able to identify alternative regimens which were predicted to be effective	206 (100%)	74 (100%)
(2) No. (%) of category 1 alternatives with a higher estimated probability of response than those of the regimen used in the clinic	175 (85%)	65 (88%)
(3) No. (%) of category 2 alternatives where one or more of the alternatives were less costly than those of the regimen used in the clinic	175 (100%)	65 (100%)
(4) Mean number of alternatives in category 3	10	8
(5) The mean cost saving from the median costing alternative in category 4 for each patient (95% CI)	$364 ($332, $395)	$421 ($361, $481)
(6) The mean percentage cost saving of the above	41% (38%, 45%)	45% (38%, 51%)
(7) The mean cost saving of the least expensive option from category 3 for each patient (95% CI)	$555 ($509, $601)	$638 ($554, $723)
(8) The mean percentage cost saving of the above	63% (61%, 65%)	68% (64%, 71%)
